# Supplementary material for: Migrant healthcare workers during COVID-19: bringing an intersectional health system-related approach into pandemic protection. A German case study
Source: Front Public Health. 2023 Jul 18;11:1152862. doi: 10.3389/fpubh.2023.1152862 (PMC10393282; doi:10.3389/fpubh.2023.1152862)
Supplement: Supplementary file 1 [file Presentation_1.PPTX]

## Slide 1
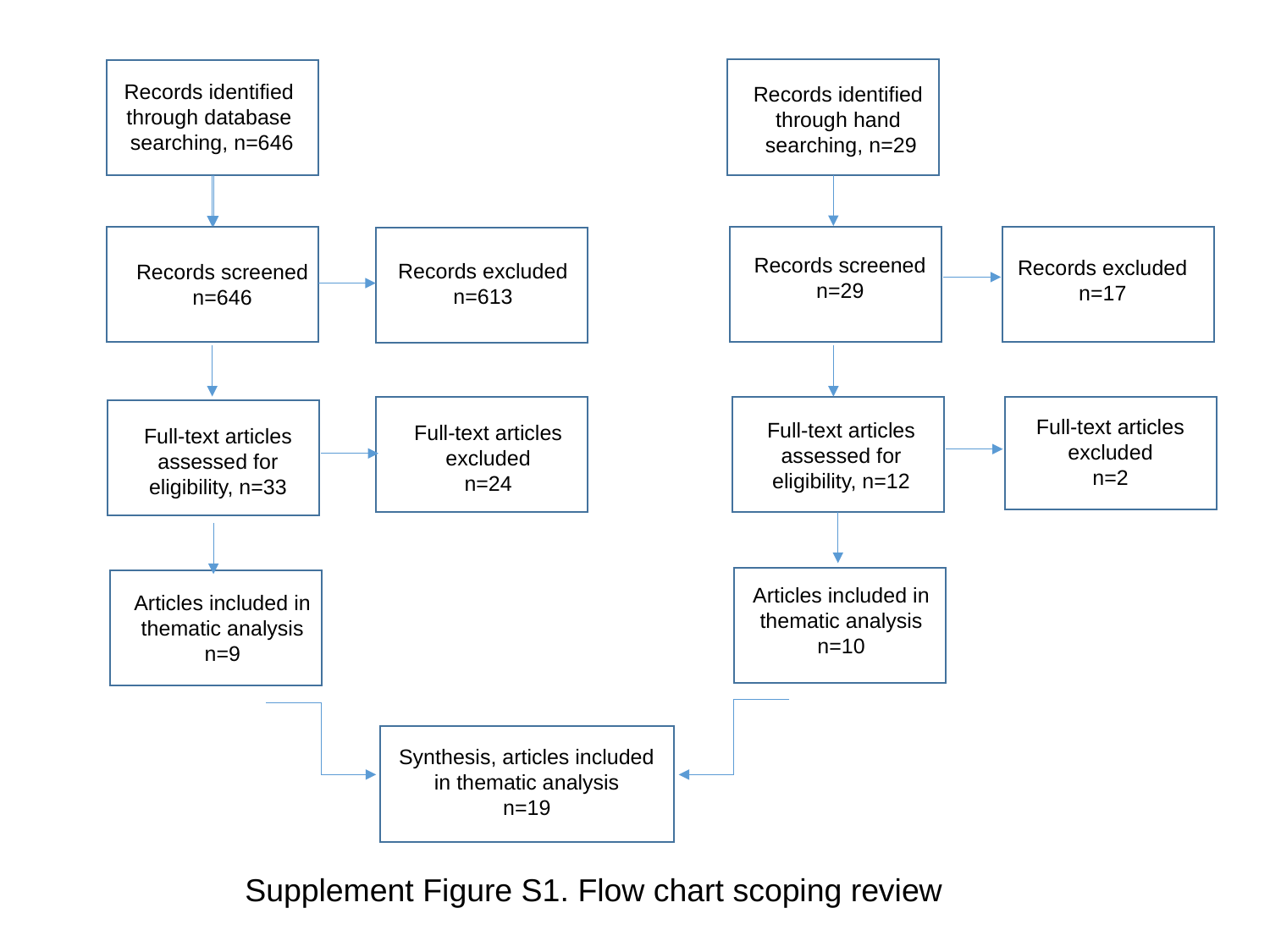

Records identified
through database
searching, n=646
Records identified
through hand
searching, n=29
Records screened n=29
Records excluded
n=17
Records excluded n=613
Records screened
n=646
Full-text articles excluded
n=2
Full-text articles assessed for eligibility, n=12
Full-text articles excluded
n=24
Full-text articles assessed for eligibility, n=33
Articles included in thematic analysis
n=10
Articles included in thematic analysis
n=9
Synthesis, articles included in thematic analysis
n=19
Supplement Figure S1. Flow chart scoping review
